# Neonatal lupus erythematosus—a rare syndrome of transient autoimmunity

**DOI:** 10.1002/ccr3.6004

**Published:** 2022-06-24

**Authors:** Nikolina Zdraveska, Aco Kostovski, Aspazija Sofijanova, Snezana Jancevska, Jana Jovanovska, Milena Kacarska, Katerina Damevska

**Affiliations:** ^1^ University Children Hospital, Faculty of Medicine Ss Cyril and Methodius University in Skopje North Macedonia; ^2^ University Clinic for Obstetrics and Gynecology, Faculty of Medicine Ss Cyril and Methodius University in Skopje North Macedonia; ^3^ University Clinic of Dermatology, Faculty of Medicine Ss Cyril and Methodius University in Skopje North Macedonia

**Keywords:** antinuclear antibodies, lupus erythematosus, neonatal lupus erythematosus, skin lesions

## Abstract

Neonatal lupus erythematosus (NLE) is a rare autoimmune disease due to a passive transfer of maternal autoantibodies to the fetus. The clinical spectrum is variable and includes skin lesions, cardiac, hematological, or hepatobiliary disorders. We report an NLE case presenting with skin eruption that was initially considered as tinea.

## Abstract 2

Neonatal lupus erythematosus is a rare neonatal disease due to maternal autoimmunity which can have various clinical presentations. Although cutaneous NLE is a transient condition, it should be recognized promptly due to the risk of recurrence in the subsequent pregnancies.

## 1 INTRODUCTION

Neonatal lupus erythematosus (NLE) is an uncommon acquired autoimmune disease caused by the passive transfer of maternal autoantibodies including anti‐Sjögren's syndrome A (Ro/SSA), anti‐Sjögren's syndrome B (La/SSB), ribonuclear protein (RNP), and antiphospholipid (APL) antibodies.^1^ Seropositive mothers usually have known or undiagnosed autoimmune diseases at the time of childbirth. The clinical spectrum is variable and includes skin lesions, cardiac, hematological, or hepatobiliary disorders. Skin eruptions are common in NLE. However, they are often misdiagnosed with other erythematous rashes that are frequently seen in the neonatal age such as skin infection or eczema, particularly if the mother is asymptomatic.^2^ It is considered that more than half of seropositive mothers are completely asymptomatic and become aware of these antibody reactivities upon detection of fetal bradyarrhythmia or neonatal skin rash appearance.^2^ We report a case of a female newborn referred for evaluation of annular rash on the face and trunk that was initially considered as tinea. Differential diagnoses of NLE, evolution of lesions, management, and prognosis are discussed.

## 2 CASE REPORT

**FIGURE 1:**
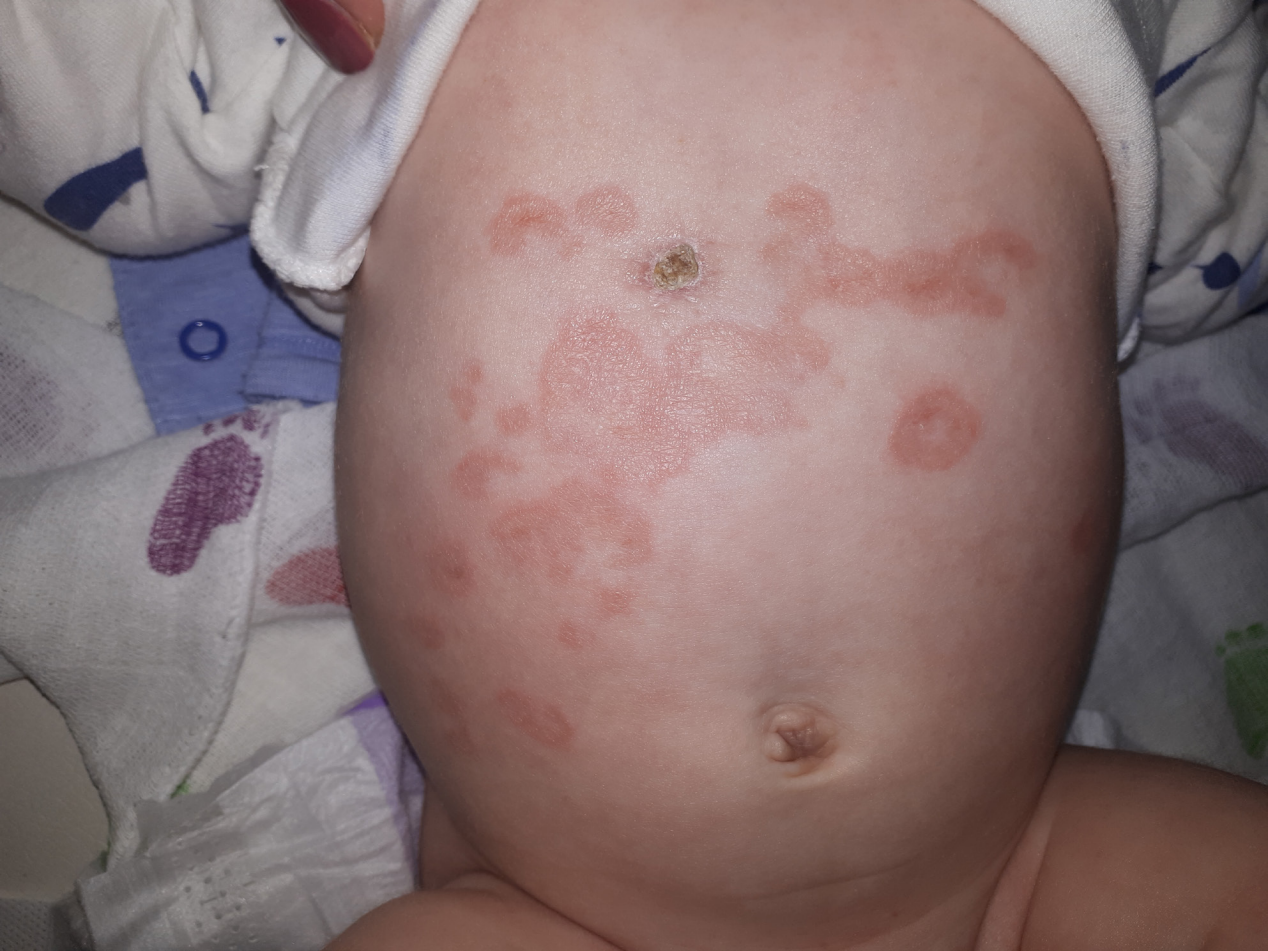
Erythematosus eruption on the trunck

**FIGURE 2:**
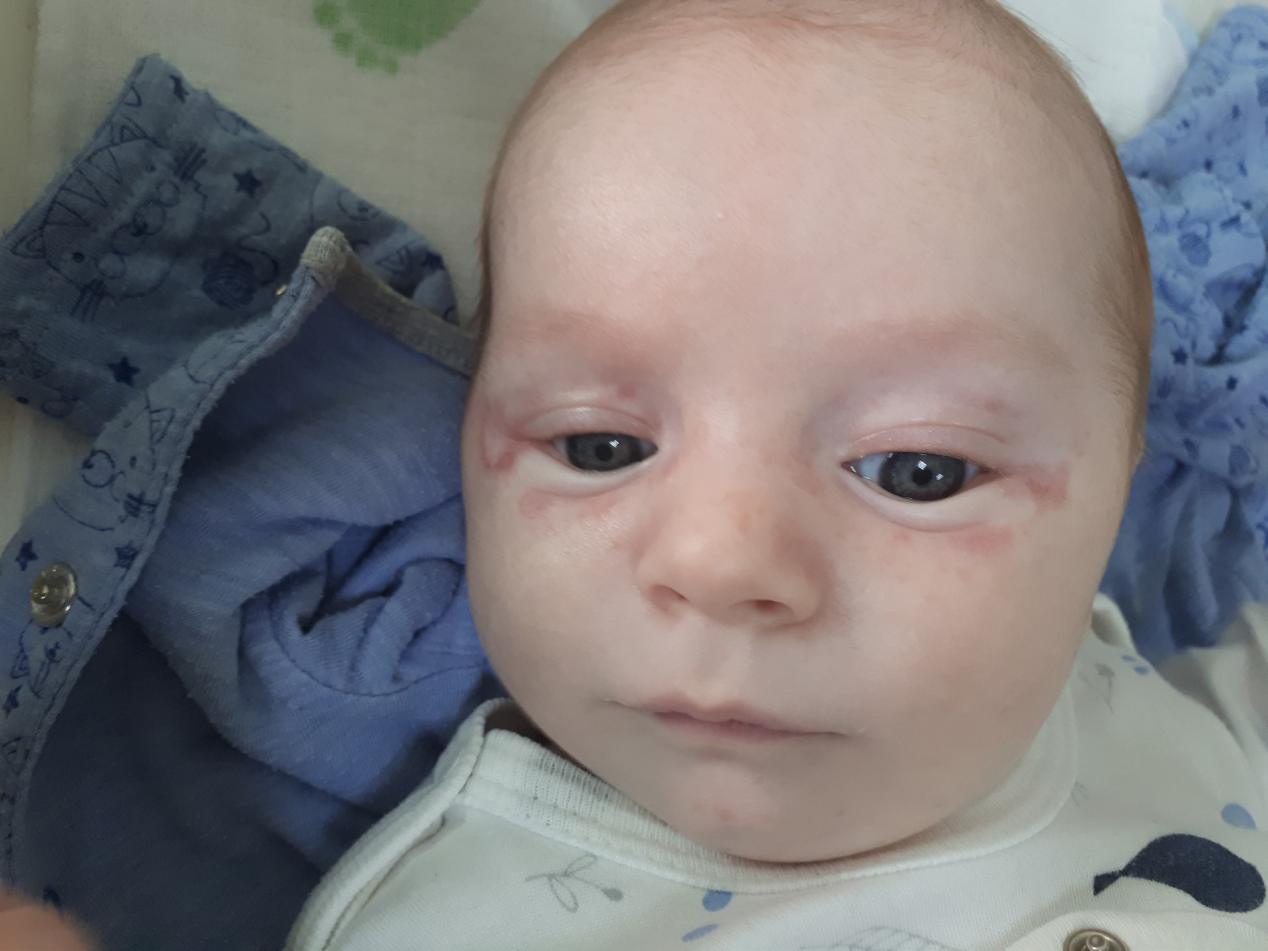
Annular and discoid patches with central hypopigmentation on the face

A 3‐week‐old female newborn was referred for evaluation of a rash on the face and trunk initially considered as tinea. The rash had been present since 2 weeks of age and the lesions were increasing in size over the last week. Otherwise, the child appeared well and was gaining weight adequately. The baby was born at term, by a spontaneous delivery to a 26‐year‐old healthy gravida 2 para 1 women. The mother had a spontaneous abortion prior the actual pregnancy. She received routine prenatal care with all prenatal laboratory and fetal ultrasound findings within normal limits. Birthweight was 3060 g, and birth length was 50 cm. Apgar score was 8 and 9 in the first minute and the fifth minute, respectively. In the maternity hospital, the baby received antibiotic treatment for nonspecific neonatal infection and was discharged on the 5th postnatal day with mild thrombocytopenia that was considered transient. Physical examination revealed skin and mucous membrane paleness and multiple annular lesions with central hypopigmentation, with dimensions 1–3 cm, solitary or conflating, mainly localized on the face, the anterior side of the chest, and abdomen (Figures 1, 2). There were no signs of inflammation or pathologic discharge. Heart rate was rhythmic; the frequency was 150/min, without any pathological murmurs. The abdomen was soft and non‐tender with no hepatosplenomegaly. Initial laboratory analysis showed anemia, thrombocytopenia (Hgb 90 g/L, RBC 2.88 × 10^12^/l, PLT 58 × 10^9^/l), and elevated serum transaminases: AST 216 U/L (normal 15–59); ALT 136 U/L (normal 9–72). The serum electrolytes, protein, albumin, blood urea nitrogen, and creatinine levels were normal.

Histological examination showed lamellar orthokeratosis, a completely flattened epidermis with vacuolar degeneration in some cells on the basal cell layer. There was massive edema in the superficial and the deeper dermis, disjunction of collagen and elastic fibers, and intense lymphocyte inflammatory infiltrate with interface distribution on some sides. Further serologic studies showed positive anti‐Ro/SSA (189 U/mL, normal <20) and anti‐La/SSB (240 U/mL, normal<20) antibodies. All the other antinuclear antibodies were negative, as follows: anti‐dsDNA<10 U/mL (normal<30); nRNP/sm 3.58 U/mL (normal <20); anti‐Ribosomal P protein <2.09 U/mL (normal <30); anti‐nucleosomes <2.0 U/mL (normal <20); and anti‐histones 4.95 U/mL (normal <30). In addition, the maternal serologic screen for antinuclear antibodies was positive for anti‐Ro/SSA and anti‐La/SSB, with values of 263 U/mL and 293 U/mL, respectively. Based on the above finding, the diagnosis of NLE was made. Symptomatic treatment was provided, and hydrocortisone cream was applied on the lesions. During the follow‐up, hematologic and liver tests normalized at 3 onths of age, followed by clearance of cutaneous lesions and negative serology at 7 months of age.

## 3 DISCUSSION

Our case fulfilled the criteria for diagnosis of cutaneous NLE accepted by the most of authors in the literature including typical lesions in the first year of life, histologic evidence of basal cell vacuolar degeneration, and mononuclear cell infiltration, and positive anti‐Ro/SSA or La/SSB.

These maternally transferred autoantibodies mediate fetal autoimmune disease through the formation of antibody complexes with apoptotic antigens in the skin, liver, or heart.^3^, ^4^ It is a rare condition as only 1%–2% of infants with positive maternal autoantibodies develop NLE. Conversely, about half of women with circulating autoantibodies who have children with NLE are completely asymptomatic; however, they might develop some rheumatologic disease later in life.^5^

NLE presents mainly with cardiac, dermatologic, hepatic manifestations and less commonly with hematologic, central nervous system, or splenic abnormalities. Its most serious complication is the atrioventricular block, which can be diagnosed in utero.

Skin lesions are common in NLE and may be present at birth or several weeks after birth. The morphology is similar to the subacute cutaneous SLE rash characterized by round, discoid, or elliptical erythematous patches or plaques with central clearing, with or without fine scale. The neonatal lupus lesions typically involve the face, scalp, neck, trunk, and extremities, and ultraviolet light (sunlight or phototherapy) frequently induces or exacerbates them. Telangiectasia is described in some cases and is considered to be the result of angiogenesis disturbances.^6^, ^7^

The histopathology of the skin lesions is typical for epidermal necrosis, basal cell vacuolar degeneration at the dermo‐epidermal interface, and adnexal structures. Additionally, urticaria‐like lesions with superficial and deep perivascular and periadnexal lymphocytic infiltrates may be present.^8^

The neonatal lupus rash remits within the first year in almost all cases in parallel with the clearance of maternal antibodies. Mild epidermal atrophy, telangiectases, and dyspigmentation may persist further, particularly if the skin lesions were highly inflammatory. Skin manifestation of NLE should be differentiated from various erythematous rashes seen in the neonatal period such as annular urticaria, tinea corporis, eyelid telangiectasias, erythema multiforme, congenital rubella, and congenital syphilis.^6^ Newborns with tinea corporis have similar skin lesions that are usually more inflammatory, as well as skin lesions in other family or contact case. In telangiectasias, there are capillary malformations on the whole skin over, not scaly, rarely multiple and tend to improve during the first weeks of life.

Treatment of NLE that affects the skin, blood, spleen, or liver is only supportive, as it is usually self‐limited and resolves without intervention over time.^9^ For skin manifestations, topical corticosteroids have been previously evaluated; however, the efficacy has not been established. Avoidance of sun or ultraviolet light exposure to prevent or minimize the rash and residual skin abnormalities is recommended.^9^

The prognosis of NLE is dependent mainly on cardiac involvement; the reported mortality rate is 20% in neonates with heart block despite pacemaker implantation. Children who have had NLE may be at increased risk of developing an autoimmune/or rheumatic disease. However, there were no babies with NLE nor in their siblings who developed SLE after long‐term follow‐up.^10^

On the contrary, the risk of NLE occurrence in subsequent pregnancies is up to 50% and the mothers should be carefully monitored with serial fetal echocardiograms.^11^

In conclusion, NLE presents a rare neonatal disease due to maternal autoimmunity, which can have various clinical presentations. Although cutaneous NLE is a transient condition, it should be recognized promptly due to the risk of recurrence in the subsequent pregnancies with more severe presentation and there is a need for careful follow‐up.

## AUTHOR CONTRIBUTIONS

NZ had main contribution in literature search, writing and drafting the manuscript. NZ, AS, SJ, JJ, and MK contributed in the diagnosing, treatment, and follow‐up of the patient. AK was consultant regarding hepatic involvement. KD performed and interpreted the skin biopsy. NZ, AK, AS, SJ, and KD edited the manuscript. All authors approved the final version.

## CONFLICT OF INTEREST

The authors declare that they have no conflicts of interest**.**

## CONSENT

Written informed consent was obtained from the patient's parent to publish this report in accordance with the journal's patient consent policy.

## Data Availability

The data that support the findings of this study are available on request from the corresponding author.
